# Supplementary material for: Viral Co-Infections of Warthogs in Namibia with African Swine Fever Virus and Porcine Parvovirus 1
Source: Animals (Basel). 2022 Jun 30;12(13):1697. doi: 10.3390/ani12131697 (PMC9265065; doi:10.3390/ani12131697)
Supplement: Supplementary file 1 [file animals-12-01697-s001.zip › animals-1787391-supplementary.pdf]

Table S1 - The CVR sequences of the warthog samples are presented together with CVRs of previously sequenced Namibian isolate

Isolate name	Species	Localities	Years	N. of repeats	CVR Tetrameric Repeat Sequences (TRS)
Nam_4593	Swine	Grootfontein	2018	24	BNAAAFBTDBNAFNBTFNBNAAAF
Nam_5538	Swine	Gobabis	2018	24	BNAAAFBTDBNAFNBTFNBNAAAF
Nam_1025_1	Swine	Windhoek	2018	8	BNAAHAAF
Nam_1028	Swine	Windhoek	2018	8	BNAAHAAF
Nam_2133	Swine	Windhoek	2018	18	BNAFNBNAAAFNBNAAAF
Nam_W06	Warthog	Windhoek	2019	24	BNAAAFBTDBNAFNBTFNBNAAAF
Nam_W17	Warthog	Windhoek	2019	20	BNAAAAAADBNAFNBNAAFA

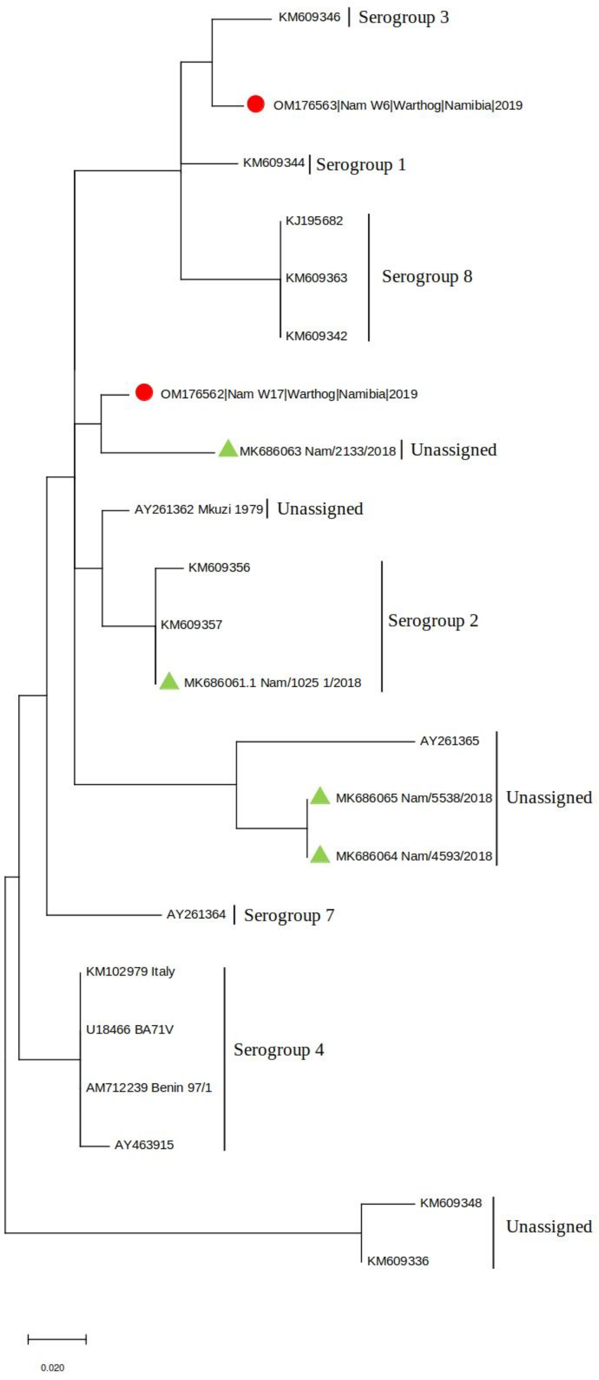

Figure S1: Neighbor-joining phylogenetic tree based on the CD2v sequence dataset
